# Population imaging of internal state circuits relevant to psychiatric disease: a review

**DOI:** 10.1117/1.NPh.12.S1.S14607

**Published:** 2025-01-28

**Authors:** Sophia Arruda Da Costa E. Silva, Nicholas J. McDonald, Arushi Chamaria, Joseph M. Stujenske

**Affiliations:** a University of Pittsburgh, Department of Psychiatry, Translational Neuroscience Program, Pittsburgh, Pennsylvania, United States; b University of Pittsburgh, Kenneth P. Dietrich School of Arts and Sciences, Pittsburgh, Pennsylvania, United States; c University of Pittsburgh, Center for Neuroscience, Pittsburgh, Pennsylvania, United States; d University of Pittsburgh, Department of Bioengineering, Pittsburgh, Pennsylvania, United States

**Keywords:** calcium imaging, internal states, neural populations, psychiatric disease

## Abstract

Internal states involve brain-wide changes that subserve coordinated behavioral and physiological responses for adaptation to changing environments and body states. Investigations of single neurons or small populations have yielded exciting discoveries for the field of neuroscience, but it has been increasingly clear that the encoding of internal states involves the simultaneous representation of multiple different variables in distributed neural ensembles. Thus, an understanding of the representation and regulation of internal states requires capturing large population activity and benefits from approaches that allow for parsing intermingled, genetically defined cell populations. We will explain imaging technologies that permit recording from large populations of single neurons in rodents and the unique capabilities of these technologies in comparison to electrophysiological methods. We will focus on findings for appetitive and aversive states given their high relevance to a wide range of psychiatric disorders and briefly explain how these approaches have been applied to models of psychiatric disease in rodents. We discuss challenges for studying internal states which must be addressed with future studies as well as the therapeutic implications of findings from rodents for improving treatments for psychiatric diseases.

## 1 Introduction

It has become increasingly clear that distributed neural populations, rather than individual neurons, mediate complex neural computations.^1^ Therefore, understanding how individual neurons in the mammalian brain work together to achieve tasks such as decision-making, cognition, and action selection requires approaches that allow for capturing population activity. There have been recent advances in technology for tracking neural activity that has greatly expanded our capability to capture activity in genetically defined populations and simultaneously record activity in many individual neurons. In this review, we will focus on imaging-based techniques, which are particularly suited for careful characterization of large neuronal populations, and we will summarize their advantages in comparison to electrophysiological approaches, which are complementary. We will then review how state-of-the-art techniques for studying neural populations have advanced our understanding of the emotional circuits dysfunctional in psychiatric diseases.

Psychiatric diseases almost ubiquitously involve dysregulated emotional responses.^2^ The study of the neural basis of emotions has been approached in animals by focusing on prototypical internal states.^3^ An internal state is a state of the central nervous system, influenced by both bodily interoception and external stimuli, which predisposes to a consistent set of somatic, physiological, behavioral, and cognitive responses. Thus, an internal state is the combination of a brain and body state that are triggered for adaptation to a changing external environment. We share the view of Anderson and Adolphs, as presented in their 2014 review outlining a framework for studying emotions in animal models, that though internal states in animals may not be directly homologous to categories of human emotions (e.g., rodent defensive states to “fear”), internal states share “emotion primitives,” the building blocks from which more complex emotional states are constructed that organize motivated behavior.^4^ Human emotional states are more complex in that they also incorporate the conscious experience of that emotion, adding a cognitive component to human emotional states absent in animals, which lack a theory of mind.^5^^,^^6^

Nevertheless, animal models can help us understand, from an evolutionary perspective, the primitive brain states that are necessary for these complex emotions to arise.^4^ Deciphering how internal states are represented in the brain is critical for developing interventions for psychiatric disorders, which are hypothesized to involve dysregulation of internal states that manifest in maladaptive behaviors.^3^^,^^4^^,^^7^ By understanding the neural mechanisms that govern these states, researchers can pinpoint potential therapeutic targets and devise strategies to restore normal function.^8^^,^^9^

Over the past two decades, fluorescent imaging, especially calcium imaging, has emerged as a critical tool for tracking single-cell activity within neural circuits.^10^ Historically, research predominantly focused on the activation of individual neurons or small groups of cells. Yet, it has long been theorized that populations of coordinated neurons, activated in temporal sequence, are the fundamental unit underlying neural computations and plasticity.^11^ This idea was suggested in 1949 by neuropsychologist D. O. Hebb, who proposed the existence of cell assemblies, networks of neurons that are transiently and synchronously activated during repetitions of the same experience.^12^ Over the last two decades, technological advancements have made it possible for us to simultaneously track the activity of large neuronal populations, providing support for the existence of cell assemblies underlying motivated behavior,^11^^,^^13^ and providing other profound insights into the coordination and function of neural circuits.^4^^,^^14^ This shift has been crucial for understanding how large ensembles of neurons coordinate complex behaviors and internal states.^3^^,^^4^^,^^15^^–^^17^

This review aims to summarize the advancements of *in vivo* single-cell imaging technology and the insights it has provided into the study of internal states. We will discuss how these imaging techniques have advanced our understanding of the neural circuits that subserve internal states and the implications of these findings for understanding and treating psychiatric disorders. This review will focus on two major internal states, appetitive and aversive, which are applicable to a wide range of psychiatric diseases. We will then discuss how these techniques have provided direct insight into our understanding of psychiatric disease.

## 2 Single Cell Imaging Methods

Here, we will briefly summarize population imaging techniques, which are well established and extensively reviewed elsewhere,^18^ focusing on the scientific advancements that the technology has permitted over electrophysiological methods to underscore the extended capabilities provided by state-of-the-art developments described in the next section. The imaging of single-neuron activity is achieved using calcium or voltage-sensitive fluorophores. Initially, exogenously applied dyes were applied to the brain,^19^ but activity-dependent fluorophores can now be expressed genetically in transgenic animal lines^20^ or by viral expression using neurotrophic viruses,^10^^,^^21^^–^^23^ especially adeno-associated vectors which exhibit low toxicity and long-lasting expression in mammalian cells.^24^ Most single-neuron imaging uses calcium-sensitive indicators such as the various generations of GCaMP fluorophores,^10^^,^^22^^,^^23^^,^^25^^–^^27^ but genetically encoded voltage-sensitive indicators are also increasingly used, as they have been rapidly improved for *in vivo* use over the last decade.^28^^–^^32^ Genetically encoded neurotransmitter indicators are also used, and the repertoire of available indicators is being rapidly expanded^33^^–^^35^ (recently reviewed elsewhere^36^). Calcium-sensitive indicators are brighter and easier to image than voltage-sensitive indicators owing to their cytosolic localization, whereas there are inherent constraints on the brightness of voltage indicators due to voltage changes being spatially restricted to the cell membrane (Please see review by Bando et al.^37^). Nevertheless, voltage-sensitive indicators have dramatically improved temporal resolution, directly reflect neuronal excitation, and can capture subthreshold activity, making them invaluable tools that are complementary to calcium indicators.

Imaging-based approaches offer multiple advantages over electrophysiology for the understanding of neural populations at the cost of a few disadvantages. First, imaging approaches permit restricting expression to particular genetically defined cell types using transgenic mouse lines and viruses.^38^ In one common strategy, mouse lines express an enzyme, cre-recombinase, under a specific promoter that restricts its expression to a subset of cells.^39^ Although cre can be used for conditional knockout of genes flanked with loxP sites, it is primarily used for calcium imaging to invert genetic sequences flanked by double loxP sites so that genes are flipped into an active configuration; this strategy is termed double-foxed inverted open reading frame (DIO) or FLEX.^40^ Cre-expressing mice can be crossed to transgenic mouse lines with cre-dependent genetic sequences or injected with cre-dependent viruses to drive fluorophore expression. Likewise, cre can be virally expressed, and thus, trans-synaptic viruses can be used to label cells based on their retrograde or anterograde connectivity.^41^

This approach can be expanded to use other similar recombinases such as flp for more complex expression strategies. When cre and flp are expressed in different cell populations, they can be simultaneously studied by cre-dependent and flp-dependent expression of calcium-sensitive fluorophores of different colors (typically green and red).^42^ Further, fluorophore expression can be targeted using intersectional viruses whose expression is dependent on two or more different recombinases.^43^ We will briefly explain intersectional strategies with flp and cre, but more complex strategies can utilize other recombinases such as Vcre.^44^ Using cre and flp together, boolean logic can be applied to genetic expression: Cre on–Flp on, Cre on–Flp off, or Flp off–Cre on.^45^ Thus, unique combinations of genetic expression can be achieved. This is useful when cell populations are not uniquely defined by one gene but are uniquely defined by the presence or absence of a second gene. For instance, in the first example of its use, Taniguchi et al. demonstrated that 20 classes of GABAergic neurons could be uniquely labeled and studied using a combination of cre and flp expression driven under different promoters.^43^ Another important application involves labeling afferent and efferent neurons and defining di-synaptic pathways based on cre and flp expression.^46^

In another strategy, calcium-sensitive fluorophores are expressed pan-neuronally and then static fluorophores of a different color are expressed in a cre-dependent manner, such that cre-expressing cells are co-labeled.^47^ In theory, multicolor approaches could permit co-labeling with more than two colors, but blue and infrared fluorophores remain relatively dim and/or liable to photobleaching,^48^^,^^49^ limiting their use *in vivo*. The rapid development of these tools suggests that it will not be long before new, improved fluorophores are developed that overcome these limitations and only further expand the capabilities of population imaging technology.^21^

Another advantage of imaging-based approaches is dramatically improved spatial resolution. Although cells recorded by electrodes can be known to be within a certain spatial radius, the spatial localization capacity will be an order of magnitude or two worse than single-cell imaging. Although quantitative spatial resolution calculations for individual electrodes rely on electrode impedance and cell size (among other considerations), Zhang et al.^50^ found that the widely used Neuronexus 1.0 was able to resolve spikes within a 100  μm radius of the shank within the macaque visual cortex. Single electrodes or electrode bundles thus offer very limited spatial information, but spatial localization can be improved with single-shank silicon probes, which include closely spaced electrode contacts through depth, and multi-shank silicon probes, which further expand electrode contacts in an orthogonal axis.^51^ Thus, within the axis of the shank, spatial resolution can be as little as tens of microns depending on electrode spacing,^42^ whereas the spatial resolution in the multi-shank axis will be limited by shank spacing, and in practice will be on the order of several hundred microns or more, though recent innovations may permit reducing this separation with state-of-the-art arrays to 66  μm among shanks.^52^ With cellular resolution, imaging-based approaches can localize individual cells within a two-dimensional plane. Cellular fluorescence signals may still overlap due to point spread function expansion in the axial direction and strong fluorescence in neurites, and this effect can be limited by restricting the expression of calcium-sensitive indicators to the soma.^53^ This increase in spatial resolution of imaging-based approaches greatly aids in tracking the same neurons across multiple days.^54^^,^^55^ This can also be achieved with silicon probes, but this is more technically challenging,^56^ and the confidence that the exact same cells are being recorded is lower due to reliance on spike waveforms, rather than having many three-dimensional imaging landmarks to aid cellular localization with imaging.^57^

Further, imaging-based approaches can achieve even higher resolutions *in vivo*, allowing for imaging of subcellular compartments such as dendrites and axons. Although dendritic recordings may be achievable electrophysiologically with *in vivo* intracellular patch clamp, this approach remains technically challenging, especially to patch more than one dendrite at a time.

Both imaging and electrophysiological approaches can be combined with optogenetic approaches for the manipulation of neural activity while tracking cellular activity.^58^^,^^59^ In brief, optogenetics utilizes light-sensitive channels or pumps that either excite or silence cells that express them.^60^ As with fluorescent indicators, these can be expressed virally or via various transgenic rodent lines. When used with electrophysiology, light for optogenetic stimulation is typically emitted from a fiber optic that is affixed to the electrodes^61^ or via micro-LEDs embedded within the shank of a silicon probe.^62^ These approaches can be used to activate large brain regions or focal spots, but the light cannot be restricted to the level of individual cells. On the other hand, high repetition infrared lasers, such as those used for two-photon imaging (but typically with higher power and lower repetition rate) permit targeting individual neurons or neural populations.^55^ Thus, a population of interest can be identified with two-photon calcium imaging, and then, these cells can be specifically targeted for two-photon excitation. There are nuances to the options for achieving this excitation, including resonant-galvo-galvo (RGG) scan heads, random access scanning, and holography, which are beyond the scope of this review. Please see Mahmoudi et al.^58^ for a thorough review of state-of-the-art multiphoton stimulation technology. Thus, two-photon calcium imaging approaches open an avenue into the specific manipulation of functionally defined populations of neurons. This technology is still nascent and under rapid development, but a few findings achieved with this technology are discussed below.

The major disadvantage of calcium imaging-based approaches are their temporal resolution and cost (particularly for multiphoton microscopy) and the limited ability to capture the timing of individual spikes. The temporal resolution of calcium-sensitive sensors for predicting action potentials is limited by two factors: (1) intracellular calcium dynamics associated with individual neuronal depolarizations and (2) the specific kinetics of individual calcium indicators. In the simplest scenario, calcium enters a depolarized cell via voltage-gated calcium channels, which lead to calcium transients, whose decay has been approximated to be on the order of ∼80  ms in principal cell bodies^63^ and ∼15  ms in dendrites near the cell body.^64^ Calcium dynamics can be complicated by intracellular calcium release and calcium propagation within neurons (see Ross [2012] for a review^65^). Calcium indicators themselves have a variable fluorescence decay due to their calcium binding dynamics. For single-action potential detection with excellent signal to noise, it is only the dynamics of calcium influx that should determine spike detection accuracy because these can be detected by rapid increases in signal as calcium influx has been found to be nearly instantaneous with action potential occurrence,^66^ whereas decays are then ignored. However, there is inherent noise in imaging, so if the decay constant is very large, this may influence the ability to detect the timing of individual activations.^66^ GCaMP proteins are among the most commonly used calcium sensors and have been developed over multiple generations, with improvements in temporal dynamics, particularly with regard to activation time course.^21^^,^^23^^,^^26^^,^^27^ The current generation jGCaMP8 family exhibits activation kinetics of 5 ms half-rise times in response to a single action potential^21^ Thus, when tested with doublets, this latest generation of jGCaMP proteins was able to resolve individual spikes at frequencies up to 50 Hz.^21^

Fluorescent reporters to some extent will also have nonlinearity with spiking activity, which can impact interpretability, depending on the extent of nonlinearity. For instance, jGCaMP6s responses are highly nonlinear,^10^ whereas jGCaMP8 sensor responses exhibit substantially improved linearity between fluorescence signals and spiking activity.^22^ Overall, jGCaMP8m and jGCaMP8s outperform other sensors in terms of spike detection accuracy, including jGCaMP8f, which is slightly dimmer but exhibits the fastest fluorescence decay kinetics.^22^ Deconvolution methods have been developed to predict the likelihood of spikes given recorded calcium dynamics, and these methods continue to be rapidly innovated.^21^ The jGCaMP8 family achieves improved accuracy over the previous generations, with F-scores approaching 0.8.^22^ Nevertheless, they remain unsuitable for analyses that require knowledge of precise spike timing on millisecond time scales, and this is especially true for fast-spiking neurons, which can reach instantaneous rates of greater than 400 Hz.^67^ Further, widely used spike prediction methods based on non-negative deconvolution provide an output that should not be interpreted as the timing of individual spikes,^68^^–^^70^ though there exist newer models that provide individual spike predictions^71^^,^^72^ which may overcome this limitation. Of course, voltage indicators very reliably track individual action potentials, but achieving this temporal resolution requires fast acquisition rates which may limit the volume of tissue that can be captured.^37^ Despite some limitations with temporal resolution, imaging-based approaches offer many worthwhile advantages that often outweigh the disadvantages for studies of large neural populations.

**Fig. 1:**
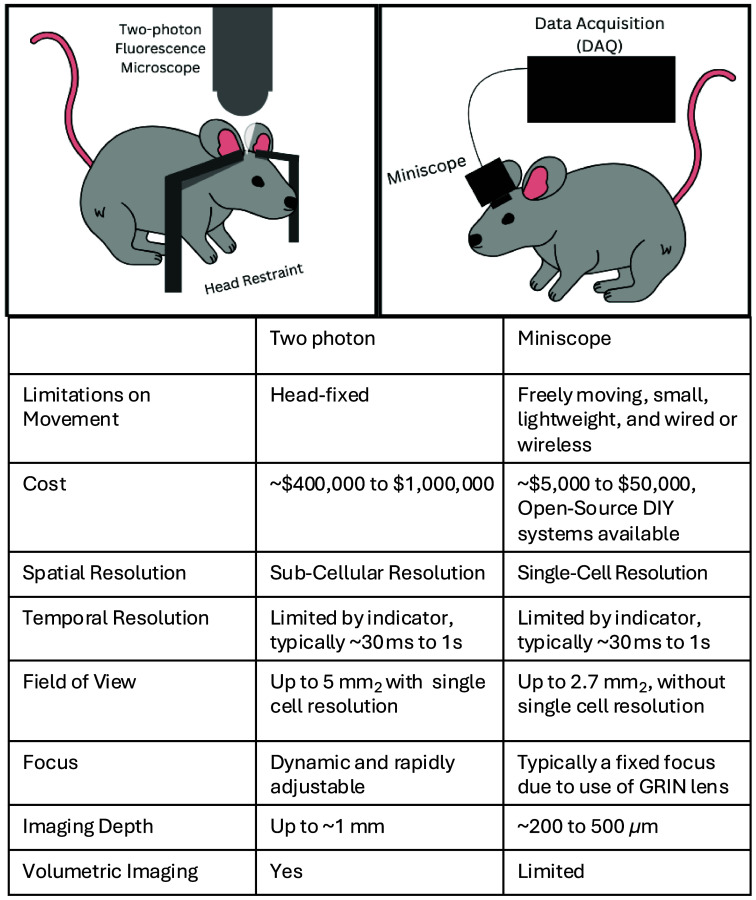
Comparison of two-photon and miniscope imaging techniques. Two-photon microscopy offers high-quality imaging but it requires rodents to be head-fixed. On the other hand, miniature microscopes (“miniscopes”) place the constraint of miniaturization on imaging capabilities to allow for freely moving applications. Although one-photon miniscopes remain the dominant technology in use, there are multiple extensions that greatly expand their capacities, as reviewed in the main text. Many recent innovations in both imaging technologies have yet to be widely adopted or commercialized. Imaging depth numbers refer to the distance below the GRIN lens for miniscopes or other optical components (e.g., microprisms). As noted in the main text, deep imaging can be achieved by using a GRIN lens and/or prisms.

Two approaches for *in vivo* imaging have come to the forefront: two-photon imaging and miniature integrated microscopes or “miniscopes” (Fig. 1). These advancements in imaging technology have led to unprecedented access into brain circuitry, allowing researchers to monitor brain activity both in freely moving mice and on timescales of days to weeks, both of which had been previously impossible.

### 2.1 Two-Photon and Multiphoton Imaging

Two-photon excitation microscopy differs from traditional confocal microscopy by releasing short, almost instantaneous bursts of infrared photons at approximately double the excitation wavelength of the fluorescent markers within the sample, rather than a constant beam at the excitation wavelength.^73^ Using this technique, fluorophores are only excited by the coincident arrival of two infrared photons, which mostly limits fluorescence to a thin optical section as two photons are less likely to arise coincidentally in out-of-focus planes. This significantly reduces phototoxicity and bleaching. Due to the lower absorbance of infrared light by tissue, deep penetration is possible (theoretically as deep as 1.4 mm in the brain can be achieved but typical applications limit the depth to ∼500 to 600  μm for high-resolution imaging^74^^,^^75^). This capability permits imaging of cortical neurons, both superficial and deep, in awake-behaving animals. When combined with an implanted gradient refractive index (GRIN) lens or prisms, two-photon imaging can also reach deeper cortical and subcortical structures, at the cost of anatomical damage to adjacent tissue.^76^^,^^77^ A particular strength of multiphoton imaging techniques is the capacity for multicolor imaging, permitting the tracking of multiple genetically defined cell populations^78^ (for a review of multiphoton imaging, please see Luu et al.^74^).

Recent developments in three- (and four-) photon microscopy have sought to push the boundaries of two-photon imaging further.^79^^,^^80^ These techniques utilize even longer wavelengths of light, allowing for deeper imaging into the brain tissue with high spatial resolution. Two- and three-photon imaging have been combined for volumetric imaging.^81^ Although these advancements are promising, their use has generally been limited to labs with extensive expertise in optics due to the higher complexity of the imaging systems. Likewise, they require the use of high light intensities which limits their speed of acquisition as lower repetition rate lasers are needed to minimize thermal damage.

Mesoscale two-photon imaging techniques have also been recently invented that greatly expand the spatial extent over which single-cell imaging is possible, including near-simultaneous acquisition in spatially distant brain structures. Among the key innovations in this area are Diesel2P, Quadroscope, and 2p-RAM, each leveraging similar techniques to greatly expand the field of view of two-photon imaging.^82^^–^^84^ They are all based on the use of large, custom objectives and scan engines that allow imaging subsections of a large field of view, up to 5 mm compared with a conventional two-photon microscope with a field of view of up to 2 mm for single-cell resolution (1 mm being most typical).

Multiphoton imaging methods have also been extended to permit volumetric imaging, either by quickly changing the focal plane either with motors or electrotunable lens (fully reviewed elsewhere^85^) or by simultaneously imaging from multiple planes using a variety of advanced techniques, including multiplexing (spatial, time-division, and/or wavelength-division) and point spread function sculpting.^86^^,^^87^ The simplest implementation of spatiotemporal multiplexing involves splitting the output of an infrared laser and then displacing those beams in space (targeting different spatial locations simultaneously) or in time (delaying one of the beams so that the photons arrive at the sample slightly later), which was first demonstrated *in vivo* by Cheng et al. in mouse barrel cortex,^88^ based on technology developed a decade earlier.^89^ More complicated multiplexing schemes, for instance, using the interference of phase-shifted laser pulses to generate amplitude modulation that permits frequency-division multiplexing,^90^ exist but are beyond the scope of this review (see Papagiakoumou et al.^85^ for more information). The complementary approach of sculpting the point spread function involves manipulating the laser beam to illuminate fewer, larger voxels, maximizing signal to noise while retaining cellular resolution for fast volumetric scanning applications.^86^

The number of innovations for volumetric imaging using these approaches over the last decade is extensive. These technologies have been recently reviewed,^91^^–^^93^ but even in the time since these reviews were published, several novel technologies have been published. We will highlight a few examples of this technology. Prevedel et al.^86^ used temporal focusing to laterally expand the point spread function in the sTeFO microscope, which could record single planes with cellular resolution at close to 160 Hz and multiple plans for volumetric imaging at 6 to 10 Hz. Weisenburger et al.^87^ subsequently combined this approach with spatiotemporal multiplexing to achieve a 1×1×1.22  mm volume imaged at 17 Hz. More extensive multiplexing has been utilized to image massive cell populations across large volumes; for instance, light beads microscopy demonstrates the capability to record about 1 million neurons over a 5.4×6×0.5  mm volume.^94^ Another approach, acoustic-based scanning technology, particularly acousto-optic deflectors, enables random-access scanning in 3D space, focusing the laser on spatially disparate regions of interest.^95^ Individual cells can be identified before an experiment and then imaging can be limited to those specific ROIs across 3D space to permit fast, volumetric imaging of cellular activity. For instance, Geiller et al.^96^ used this technology to simultaneously record from sparse interneuron populations in the hippocampus. For completeness, we note that one-photon light field microscopy is a promising technology for *in vivo* volumetric imaging, which uses microlens arrays to focus light on different focal points, but the high level of computational complexity required for processing the data has limited its experimental use in neuroscience to date (reviewed elsewhere^92^).

In summary, two-photon imaging permits a large field of view, multiplane imaging, multichannel imaging, and high spatial resolution. Despite its numerous advantages and innovations, these innovations in multiphoton calcium imaging require animals to be head-fixed during imaging sessions, which can limit the scope of behavioral studies. Thus, miniscopes, which permit freely moving imaging, have become a dominant and highly used technology within the field of neuroscience.

### 2.2 Miniature-Integrated Microscopes (Miniscopes)

Miniature-integrated microscopes, called miniscopes, provide an alternative method to permit imaging in freely moving animals by attaching a lightweight microscope directly to the subject’s head. The original miniscope developed in Mark Schnitzer’s lab in 2011 resembled a miniaturized traditional fluorescence microscope setup using a GRIN lens and weighed only 1.9 g.^97^ The original study showed the efficacy of this design as it imaged >200 motor neurons during freely moving mouse locomotion. In the past decade, rapid advancements have made utilizing these miniature systems easier and more effective. Newer lightweight and wireless systems allow for less motor impediment during freely moving behavior studies.^98^^,^^99^ The small size of these systems has permitted the ability to image multiple brain regions simultaneously through precise placement and temporal alignment.^100^ For instance, de Groot et al.^100^ developed a significantly compact and lightweight miniscope, named the NINscope, which allowed them to mount two separate miniscopes on a single animal to record interactions between the cerebellum and cortex during movement, functioning as a proof of concept for simultaneous recording. Previous field of view limitations have also been expanded with Guo et al.^101^ developing their MiniLFOV, which is able to simultaneously image more than 1000 GCaMP expressing neurons. Similarly, Zhang et al.^102^ recently developed their systematically optimized miniature mesoscope, which used diffractive optical elements to image thousands of neurons simultaneously at 16 Hz while maintaining single-cell resolution. Miniscope technology has also been modified to permit volumetric imaging across multiple focal planes.^103^^,^^104^ Others have also added simultaneous electrophysiology recording^105^ and optogenetic stimulation^100^ systems to their miniscope systems, greatly broadening how freely moving experiments can be designed and imaged.

Further advancements have extended miniscope imaging to use two-photon laser sources. Two-photon miniscope systems^106^ allow for long-term population imaging while limiting photobleaching or phototoxicity and have further expanded their capabilities of recording complex neural dynamics with volumetric imaging.^107^ Although these two-photon miniscopes were originally limited to smaller fields of view compared with their single-photon counterparts, there is ongoing work expanding the fields of view of two-photon miniscopes to sizes similar to those of single-photon systems.^98^^,^^108^ In principle, the innovations in spatiotemporal multiplexing for conventional two-photon microscopes, described above, can be applied to two-photon miniscopes, and this technology is being actively developed.^109^ Although the miniaturization of single-photon systems has made them much more affordable, with open-source materials available,^110^ two-photon systems still require the purchase of the most expensive component of multiphoton microscopes, the high repetition rate infrared laser. To date, these two-photon systems and other specialized miniscopes have not been widely used in published literature, likely due to their recent development, cost, and a lack of commercially available systems. Thus, papers relevant to this review only utilized single-photon miniscope technology.

Compared with two-photon systems, one-photon technology suffers from high levels of background fluorescence and neuron crosstalk, making highly specialized algorithms necessary for the accurate extraction of spiking activity. In 2018, Zhou et al.^111^ developed an approach called constrained nonnegative matrix factorization-endoscope (CNMF-E), which implements pre-processing to compensate for these limitations in miniscope recordings prior to implementing a previously established algorithm for extracting activity from imaged neurons, leading to improved results over previously established approaches using principal component analysis (PCA)/independent component analysis (ICA). User-friendly packages and approaches for online calcium trace extraction are based on the same underlying algorithmic framework but have made improvements in modeling background fluctuations and motion correction.^112^^,^^113^ Though signal quality remains lower for one photon compared with two-photon data, there are many examples in the literature of the same cells being tracked across multiple sessions/days of imaging.^114^

Miniscopes are an exciting, rapidly developing technology that permits studies of neural ensembles during freely moving behaviors including those relevant to decision-making, memory, reward-seeking, and social interactions.^115^ For further review on recent advancements in miniscope systems, please see Chen et al.^116^

## 3 Aversive and Appetitive States

Recent studies utilizing imaging technology to study neuronal populations have advanced our understanding of aversive and appetitive states. Overall, the findings reviewed here underscore the critical importance of understanding activity in coordinated cell assemblies, the spatial organization of neuronal circuits, and functional distinctions of genetically defined cell populations for elucidating the mechanisms of internal state regulation. The translational relevance of many of these findings for understanding psychiatric disease will be immediately evident, and we will further highlight the disease relevance of these and related findings in the subsequent section. Aversive and appetitive states will be discussed together because they engage highly overlapping brain areas.

Appetitive and aversive states have principally been studied in animal models using innately appetitive or aversive stimuli or by associating stimuli with a positive or negative outcome (classical conditioning).^3^^,^^117^ For rodents, in addition to stimuli that directly cause pain or discomfort (such as shocks or air puffs), innately aversive stimuli include bright, open spaces such as an open field or elevated plus maze, novel environments, quinine (bitter), and predators and scents from their urine.^117^^–^^119^ Innately appetitive outcomes are typically a food or sweet liquid reward. On the other hand, in classical Pavlovian conditioning, a conditioned stimulus (CS), which otherwise has no valence, becomes associated with an outcome by repeatedly pairing it with an unconditioned stimulus (US, such as shock or sweet liquid delivery^117^). In this way, responses to the CS can be compared before and after learning, permitting the specific study of valence apart from the sensory experience of pain or consuming a reward. Behavior or less commonly, physiological changes, are monitored in these assays and used as an index of the internal state of the mice.^3^

Studies of appetitive and aversive states have centered on a subset of brain regions including the ventral tegmental area (VTA), insular cortex (IC), amygdala, medial prefrontal cortex, hippocampus, hypothalamus, striatum, and sensory cortices.

### 3.1 Ventral Tegmental Area

Our understanding of the functional properties of the VTA has been greatly enhanced by recent population imaging studies. It has been established that both rewarding and aversive stimuli activate dopaminergic VTA neurons, which then release dopamine throughout the brain, leading to plastic changes that support learning.^120^ VTA neurons have been shown to encode a “prediction error” that informs the predictability of outcomes, such as rewards.^121^ Imaging VTA neurons with a miniscope revealed that when the reward is tied to another variable, such as spatial position, this prediction error can occur not only in rapid, phasic signaling but also in slower ramping activity.^122^ A recent imaging study found that alongside this encoding of prediction error, the behavioral control of an outcome also modulates ventral tegmental activity. When punishments are unavoidable, they are preferentially encoded in dopaminergic VTA projections and their downstream targets in the basal amygdala.^123^ Surprisingly, two-photon imaging revealed that VTA projections to superficial layers of the medial prefrontal cortex, which are strongly interconnected with the amygdala, do not encode a prediction error, but instead convey valence (appetitive or aversive), with a predominance of aversive encoding that develops during aversive learning.^124^ Of note, a prior study found that VTA neurons projecting to the medial prefrontal cortex selectively signal learned safe cues rather than aversive cues,^125^ so the importance of this aversive signal remains uncertain. Expanding on the encoding properties of the VTA, Howe and Dombeck et al.^126^ used two-photon calcium imaging of VTA axons to demonstrate that a subset of VTA projections to the striatum is physically active during locomotion, and these projections were largely distinct from those that encode unexpected rewards. It was subsequently found that though most VTA dopaminergic neurons are activated by reward, they simultaneously encode a range of other sensory, motor, and cognitive variables, and this encoding is spatially organized, with functionally similar neurons close to each other.^127^ In summary, population imaging approaches have greatly refined our understanding of VTA activity, revealing that it signals more than prediction error across both appetitive and aversive states and plays a multifaceted role in regulating plasticity.

### 3.2 Posterior Insular Cortex

The IC has an important role in the top-down processing of internal emotional and homeostatic perception.^128^ It has a complex neuronal organization that suggests it is involved in both coordinating autonomic responses and receiving interoceptive feedback.^129^ The posterior insula has been suggested to mediate interoception. Expanding on this model, two-photon calcium imaging of posterior insular cortical neurons revealed the simultaneous encoding of innately valent sensory stimuli, bodily states (including thirst and chemically induced malaise), and behaviors (such as freezing), with many neurons exhibiting multisensory responses.^130^ These neurons strongly encoded prolonged periods of defensiveness evoked for over an hour after fear conditioning, and increased spatial avoidance during this time was dependent on its activity during prior shocks, whereas this activity was not required for aversive conditioning. This suggests that posterior insular cells, rather than encoding unconditioned stimuli for learning, regulate long-lasting aversive states induced by acute threat. Further, imaging revealed that posterior insular cells, which encode valent sensory stimuli, can be used to reliably predict facial expressions that mice make in response to stimuli of different valences,^131^ consistent with its suggested role in interoception and state regulation.

### 3.3 Amygdala

Population imaging of activity in the amygdala has helped to elucidate the microcircuits underlying its well-established roles in aversive and appetitive conditioning. A population imaging approach is particularly powerful for understanding how neurons encode information during learning and across multiple distinct but related conditions because the same neurons can be repeatedly imaged across days. In one of the first studies to utilize calcium imaging in the basal amygdala, Grewe et al.^114^ leveraged miniscopes to track activity in the same neurons across a week, whereas mice were aversively conditioned and then this association was extinguished. They found that the basal amygdala exhibits population encoding of unconditioned and conditioned stimuli, and aversive learning entails a progressive change of CS representations toward resembling the US pattern. Likewise, extinction involved a change away from this pattern, but toward a new representation distinct from the representation prior to learning. A subsequent study replicated this finding and demonstrated the same process for appetitive stimuli and found that during reversal learning, neural representations could be retransformed to resemble the opposite valence US.^132^ Thus, consistent with a longstanding theory of basolateral amygdala function, plastic changes during learning reorganize basal amygdala neural population responses such that conditioned stimuli directly activate patterns of activity encoding aversive outcomes, and there is a long-lasting effect on population activity, even after extinction.

Krabbe et al.^133^ used miniscopes, together with optogenetic manipulations and electrophysiology, to demonstrate an amygdala circuit mechanism that gates the finding of CS associations with aversive outcomes. They showed that vasoactive intensive peptide expressing interneurons (VIP IN) are preferentially activated by unconditioned stimuli. As in the cortex, VIP IN in the amygdala was shown to primarily target other inhibitory interneurons, and their activity serves to disinhibit excitatory cells. Finally, VIP IN activity was required for learning CS-US associations, suggesting that VIP IN activity may gate the previously observed plastic changes at the population level. Interestingly, the authors found that VIP IN themselves exhibited a similar plasticity, such that their activity diminished as CS-US associations became expected, but interestingly, silencing their activity during fear retrieval did not affect defensive behavior, suggesting that they are required for gating plasticity but not for maintenance of population activity binding CS and US. However, a follow-up preprint from this group shows that there may be significant functional heterogeneity of VIP IN, with at least a subset showing persistent activity during high defense states,^134^ so silencing these interneurons *en toto* may be masking an important functional role of a subset of these interneurons for maintaining threat representations.

By contrast, Stujenske et al.^135^ found that somatostatin-positive interneurons (SOM IN) in the basal amygdala had largely homogenous responses to auditory stimuli. Using two-photon calcium imaging, SOM IN was found to weakly respond to auditory stimuli prior to fear conditioning, and there was no change in their activation to fear-conditioned cues after learning. By contrast, these SOM IN were strongly and homogenously activated by learned neutral cues, gating incoming sensory information into the basal amygdala to prevent overgeneralized fear responses. Consistently, the activity of SOM IN could decode the identity of the CS after learning (paired versus unpaired), and this encoding was dependent on activity in the upstream prelimbic area of the medial prefrontal cortex. Therefore, SOM IN plays an important role in the suppression of threat responses to non-aversive cues^135^ and likely mediates top-down control of the basolateral amygdala by upstream cortical areas. Consistently, SOM IN activity imaged with miniscopes increases after extinction,^134^ suggesting that this fear-suppressing role may be engaged by both the extinction process and to prevent overgeneralization.

Further applications of calcium imaging in the amygdala have also greatly expanded our understanding of other spatially organized and genetically defined microcircuits that regulate aversive learning. For instance, specific populations of inhibitory neurons that selectively promote or oppose extinction were identified in the medial intercalated masses (ITC),^136^ which are adjacent to the basal nuclei and when ablated impair extinction learning.^137^ Greatly extending our understanding, this study revealed a previously unknown functional distinction between dorsomedial and ventromedial intercalated neurons. Using miniscopes, the authors showed that most dorsomedial ITC neurons activated in response to shocks during fear conditioning and after conditioning, a substantial minority responded to fear-conditioned stimuli. By contrast, ventromedial ITC neurons were not activated by shocks but instead by the omission of shocks early in extinction and then by conditioned stimuli only after extinction. Optogenetic experiments validated that the ventromedial ITC promotes extinction learning and retrieval, whereas dorsomedial ITC opposes extinction retrieval, and these opposing functions are mediated by mutual inhibition between the ITC clusters, which selectively silence different amygdala outputs.

Imaging approaches have also helped to refine an understanding of central amygdala circuits. A longstanding model of central amygdala organization is that the central medial nuclei control fear behavior and are under tonic inhibitory control by central lateral neurons termed “off” neurons, which can be inhibited by other central lateral neurons termed “on” neurons to trigger defensive responses.^138^ It had been proposed that the “off” neurons were Protein Kinase C (PKC)-delta-positive neurons,^139^ but Yu et al.^140^ utilized miniscopes to demonstrate that PKC-delta-positive neurons in the central lateral nucleus specifically activate the aversive US to promote fear learning. The authors showed that these neurons have a non-canonical influence on the lateral amygdala (LA), such that LA responses to the US require CeA PKC-delta neuronal activation. The data helped to fine a model of central amygdala organization, such that CeA is not exclusively downstream of the LA for conditioning, and “off” and “on” central lateral nucleus neurons are likely intermixed within both PKC-delta and somatostatin-positive neuron populations.^141^ Consistently, Kong et al.^142^ use miniscopes to report that roughly equal proportions of somatostatin-positive neurons are activated or inhibited by unconditioned stimuli (both appetitive and aversive). The majority are selectively responsive to the US based on valence, but surprisingly, a much smaller minority of cells exhibited significant responses to stimuli after conditioning. Together, these studies suggest that central amygdala neural populations are strongly engaged in encoding unconditioned stimuli to support learning, and how this function integrates with gating defensive behaviors after learning remains uncertain.

Gründemann et al.^114^ leveraged miniscopes to track the same basal amygdala neurons across multiple behavioral experiences, both during spatial avoidance assays and through multiple days of aversive conditioning. Using this approach, they found that consistent neural ensembles exhibited activity during behaviors associated with aversion across these innate and learned aversion paradigms (e.g., freezing and leaving relatively safe spatial zones) and that sensory stimuli, both conditioned and unconditioned, were represented in an orthogonal encoding axis across the ensembles. Interestingly, encoding did not substantially differ for basal amygdala projectors to the ventral hippocampus, medial prefrontal cortex, or the nucleus accumbens, suggesting that basal amygdala output may function as a brainwide broadcast of aversive information, rather than being restricted to dedicated pathways.

In contrast to the finding that basolateral amygdala (BLA) neurons encoded aversive spatial features in a way that overlapped with conditioned aversion, a follow-up study from the same group found that when comparing spatial and social exploration, there was fundamentally different neuronal encoding.^143^ During social interactions, BLA activity did not reflect the valence of interactions (aggressive or nonaggressive), but instead neural activity tracked slowly with the degree of social engagement.

Another strength of calcium imaging approaches is the ability to spatially localize individual cells with high precision. It was previously demonstrated that the anterior and posterior basal amygdala are preferentially activated by innately aversive or appetitive stimuli, respectively, based on intermediate early gene expression.^144^ Using optical imaging of individual neurons, basal amygdala neurons were confirmed to exhibit these spatial gradients of response patterns to appetitive and rewarding cues,^145^^,^^146^ though the differences were not nearly as stark and instead were most notable for highlighting the spatially distributed nature of valence encoding in the basal amygdala. Surprisingly, the spatial association was reversed for conditioned reward stimuli, suggesting that spatial organization of valence in basal amygdala neurons is learning-dependent.^145^^,^^146^ Using two-photon imaging and holographic two-photon excitation, it was found that innate appetitive-responsive and aversive-responsive neural ensembles are mostly independent populations that are mutually inhibitory, suggesting that the amygdala’s encoding of valence involves a weighted sum of individual BLA neurons.^145^

### 3.4 Medial Prefrontal Cortex

The medial prefrontal cortex has been demonstrated to be an important regulatory structure controlling both reward-seeking and defensive behavior.^147^ In particular, the dorsomedial prefrontal cortex (dmPFC), called the prelimbic cortex in the mouse, has been implicated in both supporting the expression of learned aversive associations^148^ and suppressing defensive responses when they are inappropriate.^135^ dmPFC neurons recorded electrophysiologically exhibit activity in response to innately aversive stimuli, aversively conditioned stimuli, and defensive behavior,^14^^,^^149^ but these data did not reveal how this encoding relates to aversive state regulation. Advancing on this prior work, Hallock et al. tracked the activity of dmPFC neurons throughout fear conditioning and found that neurons exhibited responses timed to episodes of freezing, and the encoding properties of individual cells changed with learning.^150^ Specifically, they found that the activity during freezing episodes increased when fear-conditioned tones were presented compared with context re-exposure alone, and this increase was lost with unilateral chemogenetic activation of ventral hippocampal inputs to dmPFC. Notably, unilateral activity had no behavioral effect, suggesting that this change was not a consequence of the behavior. Consistently, bilateral activation of ventral hippocampal projections to dmPFC diminished defensive freezing, suggesting that ventral hippocampal projections to dmPFC constrain defensive freezing by inhibiting freezing-related ensembles.

Agetsuma et al.^149^ performed two-photon calcium imaging in layer II/III of dmPFC using a microprism, revealing that dmPFC neurons responsive to unconditioned stimuli during aversive conditioning go on to serve as a hub for linking conditioned stimuli to conditioned defensive responses. Consistently, during appetitive conditioning, single-neuron imaging in multiple frontal cortical areas demonstrated a substantial amount of task-related activity.^151^ Interestingly, imaging together with optogenetic manipulation across multiple frontal areas revealed that the anterolateral motor cortex (ALM, also called M2) is particularly involved in suppressing anticipatory licking when it is inappropriate,^151^ whereas dmPFC is more strongly activated by appetitive stimuli. Two-photon calcium imaging revealed that the dmPFC has numerous task-related patterns of activity during classical appetitive conditioning, and specifically, corticostriatal neurons are strongly activated by rewards and these responses increase with learning.^152^ How the discriminatory functions of the frontal cortex relate to functions supporting aversive and appetitive conditioning remains uncertain.

### 3.5 Hippocampus

Population imaging has also elucidated hippocampal circuits encoding both innately aversive and aversively conditioned contextual features. Dorsal hippocampal area cornu ammonis area 1 (CA1) has been long implicated in contextual encoding, with “place cells” that consistently increase firing in specific spatial locations. In an early application of two-photon calcium imaging for understanding the mechanisms by which dorsal CA1 supports contextual aversive conditioning, Lovett-Barron et al.^153^ demonstrated that dendritic inhibition of CA1 pyramidal cells during unconditioned stimuli is critical for successful conditioning by preventing incorporation of the US into the context representation. A recent study using two-photon calcium imaging of hippocampal CA1 neurons found that during conditioning, these cells come to encode anticipated unconditioned stimuli (reward or shock), but only when the outcome was dependent upon the behavior of the mouse, as when a shock became avoidable.^154^ Two-photon calcium imaging of dorsal CA1 projectors to the nucleus accumbens revealed that when navigating toward a learned reward zone, they exhibit a higher rate of spatial and non-spatial task-related activity, and via conjunctive encoding are highly informative for spatially localizing the reward zone.^155^

Interestingly, it was found that upstream of CA1 in the dorsal dentate gyrus (DG), population coding also supports aversive learning. DG influences CA1 activity via a feed-forward DGàCA3àCA1 trisynaptic circuit, and it has long been implicated in differentiating novel contexts from familiar ones; for instance, optogenetic inhibition of DG can induce overgeneralization to a novel neutral context similar to a fear-conditioned one or interfere with contextual fear retrieval if a similar neutral context was experienced before fear conditioning.^156^ Seo et al.^157^ tracked activity in DG using miniscopes throughout nine days of contextual fear discrimination training, in which mice gradually learned to suppress defensive freezing in a safe context. They found that this learning was associated with an increase in the population ensemble encoding of the safe context. Further, they found that restraint stress or activating locus coeruleus chemogenetically impaired learning and blocked this shift in population ensemble encoding. They determined using pharmacological manipulation and optogenetics that this effect was driven by beta-2-adrenergic signaling on DG hilus interneurons, increasing inhibitory tone in the DG. Thus, dorsal hippocampal circuits help with binding valence to contexts during learning but are not obviously encoding appetitive or aversive states directly.

On the other hand, ventral CA1 has been long implicated in spatial avoidance behaviors, such as avoiding open, bright spaces, and has been thought to more directly mediate aversive behavior. Using miniscope imaging in ventral CA1, Jimenez et al.^158^ found that during exploration of the elevated plus maze, which consists of innately aversive open arms compared with the relatively safe closed arms, ventral CA1 but not dorsal CA1 exhibited preferential activation for the open arms. Then, using an intersectional viral approach, they were able to demonstrate that surprisingly, ventral CA1 neurons projecting to the basal amygdala did not exhibit this encoding, but instead, this was found selectively for projectors to the lateral hypothalamus.^159^ A recent follow-up preprint from the same lab again utilized miniscopes to demonstrate that ventral CA1 neurons encode specific anxiogenic contextual features,^160^ suggesting that ventral CA1 is a critical circuit hub for aversive state regulation, possibly for biasing spatial navigation away from danger.

### 3.6 Hypothalamus

Population imaging has unveiled the complex role of the hypothalamus in the modulation of threat behaviors. Lovett-Barron et al.^14^ manipulated multiple populations of hypothalamic neurons to link different classes of peptidergic neurons in the neuroendocrine hypothalamus to their roles in the rapid avoidance of homeostatic threats. They used calcium two-photon imaging in zebrafish while they were exposed to a variety of threats to their homeostasis that elicited defensive avoidance responses. The activity of genetically defined neuronal populations in the hypothalamus of behaving animals was recorded, and these cells were then identified *post hoc* using multiplexed gene expression. They classified neurons in the hypothalamus based on their responses, and surprisingly, they found that their classifications each cut across the nine genetically distinct neuropeptide-releasing populations they evaluated, suggesting that threats and responses to threats involve coordinated responses of different genetically defined hypothalamic cell populations. It was found that a subset of these cells that co-released glutamate contacted a specific premotor cell population in the brainstem which then projected to the spine to coordinate defensive motor responses.

Consistently, a subset of glutamatergic anterior hypothalamic area neurons marked by parvalbumin expression were imaged in mice using miniscopes and found to be activated in response to predatory threats.^161^ Their firing patterns remained similar after repeated exposures and anticipated future encounters with these threats. Their activation was found to active a set of brain structures involved in arousal and defensive behaviors, including the amygdala and substantia nigra, suggesting that they may be a hub for coordinating aversive internal states.

### 3.7 Striatum

The striatum, particularly the nucleus accumbens, has long been implicated in the encoding of reward and punishment, particularly for guiding motivated behavior.^162^ A few recent papers utilizing two-photon imaging to expand our understanding of the microcircuit organization of striatal activity during appetitive and aversive states, particularly demonstrating encoding of multiple related aspects of valent experiences. It was found that within striosomes, neurons are more engaged in response to reward-predicting cues, compared with matrix neurons, which are more sensitive to recently received reward history.^163^ It was found that the medial nucleus accumbens shell is responsive not only to rewards but also to the extent to which they were preferred.^164^ Using miniscopes, it was determined the nucleus accumbus core ensembles respond both to aversive stimuli and also exhibit encoding of observed aversive experiences of other mice.^165^ Interestingly, a subpopulation of neurons encoded observed and experienced stimuli oppositely, supporting the simultaneous representation of aversion and somatosensory experience.

### 3.8 Sensory Cortices

Although sensory cortices are not thought to directly regulate internal states, population imaging studies have illustrated how plasticity in sensory cortices subserves learning that relates external stimuli to internal states. In an early application of two-photon calcium imaging, Letzkus et al.^166^ identified a microcircuit in the mouse auditory cortex that integrates aversive unconditioned stimuli with conditioned auditory stimuli. They found that in anesthetized mice, foot shocks trigger disinhibition of auditory cortex principal neurons via cholinergic activation of layer 1 auditory cortical interneurons that inhibit layer 2/3 interneurons. Thus, the auditory cortex comes to relate complex auditory stimuli with aversive outcomes. Similarly, it was found that the directional tuning curves of a small population of primary visual cortex neurons with preferred orientations similar, but not identical, to an orientation paired with reward; this demonstrated a circuit mechanism by which visual generalization is induced by appetitive experiences.^167^

## 4 Population Imaging in Rodent Models of Human Psychiatric Symptoms

To illustrate how population imaging findings related to internal states hold promise for understanding the pathophysiology and advancing the treatment of psychiatric disorders, we will highlight some findings from explicit models of psychiatric disease in rodents.

### 4.1 Depression

Depressive disorders manifest with a range of symptoms, and one of the most prominent and impairing is anhedonia, a dampening of positive emotional experiences manifesting as a loss of pleasure in previously enjoyed activities. Anhedonia reflects a failure of previously rewarding stimuli to induce appetitive internal states in human beings, and thus, in elucidating circuits underlying these states, we can identify circuits likely to be dysfunctional in this psychiatric condition.

Population imaging findings have elucidated microcircuits in the hippocampus and the prefrontal cortex that regulate stress experiences and that may be targets for therapeutic intervention. Depressive-like phenotypes that resemble features of depression, including anhedonia, can be induced in mice through a variety of stress-inducing paradigms. Behavioral changes include changes in coping, avoidance, sociability, and anhedonia. Using miniscopes during chronic social defeat stress, in which mice are repeatedly attacked by aggressive, physically larger CD1 mice, it was found that the same ventral hippocampus dentate granule cells that encode innately aversive environmental features also encode stressful experiences with aggressor mice.^55^ Increasing adult neurogenesis was sufficient to dampen activity in this ensemble and block the development of depressive-like phenotypes, highlighting translational implications of understanding this population’s neural activity.

Some mice exhibit resilience after chronic social defeat, failing to demonstrate avoidance behaviors and depression-like phenotypes, and it has been of great interest to understand the neural circuit differences that typify resilience compared with vulnerability. Calcium imaging in the nucleus accumbens showed that in mice with resilient phenotypes after chronic social defeat, dopamine receptor 1 medium spiny neurons (D1-MSNs) show elevated baseline activity and stronger calcium responses during social interactions.^168^ These findings support the idea that boosting activity in D1-MSNs can promote resilient behaviors and may coordinate activity in neural circuits that support resilience.^168^^,^^169^

In another study utilizing two-photon calcium imaging, it was found that chronic corticosterone administration, which induces depressive-like phenotypes and heightens spatial avoidance, induces spine elimination in the medial prefrontal cortex, disturbing the normal pattern of correlated neural activity in prefrontal neurons.^56^ Both effects could be reversed with the administration of the rapidly activating antidepressant, ketamine, by virtue of a selective restoration of stress-eliminated spines. These findings highlight how imaging approaches can be used not only for understanding psychiatric disease processes but also for their treatment.

### 4.2 Post-traumatic Stress Disorder

Post-traumatic stress disorder (PTSD) is characterized by distressing symptoms following a traumatic event.^170^ It is widely theorized that PTSD reflects fear memories that are overgeneralized and resistant to extinction.^171^ Therefore, understanding the circuitry that is abnormally activated during fear responses in mice is crucial for gaining insights into the emotional dysregulation observed in PTSD patients. We will briefly synthesize the information provided above with relevance to PTSD pathophysiology and treatment.

Population imaging in the amygdala has revealed that aversive learning reshapes neuronal population representations to form aversive associations with contexts and stimuli.^54^^,^^114^^,^^132^ This plasticity, central to the encoding and retrieval of fear memories, is mediated by inhibitory microcircuits, which gate fear learning and expression.^133^^,^^134^ In particular, SOM IN in the basal amygdala prevents overgeneralization of fear by suppressing threat responses to neutral cues,^135^ and imaging evidence shows that these same interneurons are active during extinction,^134^ suggesting that interventions that somatostatin-positive interneuron activity could be dysfunctional in PTSD, leading to the core symptoms of overgeneralization and impaired extinction. The ventromedial ITC could be another site of dysfunction given its now-established role in promoting extinction.^136^

Likewise, the rodent prelimbic cortex, likely homologous to the human anterior cingulate cortex^172^ exhibits activity that changes with learning and encodes both aversive stimuli and defensive behaviors,^150^^,^^151^ supporting both the expression of fear^150^^,^^151^ and suppression of overgeneralization.^135^ Meanwhile, hippocampal circuits differentiate between aversive and safe contexts, with dorsal regions supporting the memory of contextual valence^153^^,^^154^^,^^157^ and ventral areas contributing to guiding defensive behavior based on contextual valence, particularly via projections to the hypothalamus,^158^^,^^160^ and regulating contextual fear learning via projections to the medial prefrontal cortex.^150^ Hypothalamic circuits have been shown to be evolutionarily conserved for modulating brain state to support arousal,^173^ and multiple neuropeptide-releasing populations there exhibit coordinated activity for mediating responses to threat, with at least part of this response mediated by glutamate co-releasing projections to brainstem premotor neurons.^14^ Thus, the encoding of aversive memories and motivated defensive responses to respond to perceived threats involve interconnected circuits in the BLA, medial prefrontal cortex, dorsal and ventral hippocampus, and hypothalamic circuits. Further, there are signatures of aversive learning in primary sensory cortices upstream of these circuits,^166^^,^^167^ suggesting that the overgeneralized fear responses seen in PTSD can involve dysfunction across multiple cortical and subcortical regions. The overwhelming stress of traumatic experiences may predispose to this overgeneralization via noradrenergic signaling, as was shown for an overgeneralization mechanism involving DG hilar interneuron activity.^157^ These insights underscore the potential for circuit-specific intervention via neuromodulation or pharmacology to improve fear discrimination and expedite the extinction of fear memories.

### 4.3 Eating and Metabolic Disorders

Eating disorders are currently diagnosed based on patterns of disordered eating that are physically impairing, induce distress, or impair functioning.^170^ Although the pathophysiology of eating disorders is complex, including components of anxiety, the sensory experience of eating, and/or body image concerns,^174^ they share a disruption of the normal relationship between the bodily need for nourishment, indicated by hunger, and interaction with food.^175^ Eating disorders have been shown to involve a loss and lack of interest in palatable foods that would have otherwise been rewarding.^176^ Population imaging approaches have enhanced our understanding of circuits mediating hunger and food consumption, with some findings that are likely related to underlying eating disorder pathophysiology.

Agouti-related peptide (AgRP) and pro-opiomelanocortin (POMC)-expressing neurons in the arcuate nucleus of the hypothalamus have been shown to be activated in states of energy deficit (hunger and thirst) and surplus/satiety, respectively.^177^ Recordings with miniscope calcium imaging revealed that the AgRP neurons active during energy deficits convey the negative valence associated with hunger and thirst, and this could serve as a teaching signal.^178^ Further, using a related technique, fiber photometry, which tracks bulk fluorescent calcium signals in a brain region using a single-optic fiber, Chen et al.^179^ demonstrated that AgRP and POMC signaling changed in response to the detection of food, even before it was consumed, suggesting that their activity regulates motivated behavior by informing about the availability of nourishment, rather than merely reflecting the energy state of the animal. Thus, AgRP and POMC hypothalamic neurons have been of significant interest in understanding the pathophysiology of metabolic and eating disorders.

Calcium population imaging approaches have not been applied yet to explicit animal models of eating disorders or obesity, but promising findings have arisen from the use of the related technique, fiber photometry, which we just discussed. It was found that states of obesity cause long-lasting desensitization of AgRP neurons to dietary fat,^180^ and in fact, exposure to high-fat foods leads to a long-lasting devaluation of standard food chow via a persistently diminished sensitivity of the negative valence signal encoded by AgRP neurons and the positive valence signal encoded by dopaminergic VTA neurons to standard food.^181^ Thus, obesity may be perpetuated by dysfunctions in brain circuits that alleviate the feelings of hunger and circuits that assign positive valence to eating. Eating in excess of homeostatic needs can be elicited in mice by contextual conditioning, and this was shown with fiber photometry and optogenetics to depend on a hippocampal projection to a specific subclass of orexinergic neurons that co-expressing somatostatin in the tuberal nucleus of the hypothalamus.^182^ These neurons responded in high-fat food-associated contexts, before food was consumed, and were required to drive excessive eating, suggesting that this hypothalamic circuit drives excessive eating based on appetitive conditioned associations.

The role of hypothalamic circuits has also been investigated to understand insufficient eating in explicit models of anorexia, namely the activity-based anorexia (ABA) model, in which rodents exercise on a voluntary running wheel while food is restricted. Results from two studies suggest that AgRP neurons regulate not only feeding but also voluntary exercise and bodily mobilization of fat stores. Fiber photometric recordings revealed that in ABA mice, AgRP neurons transiently decrease their activity at the end of voluntary exercise, resembling decreases associated with sensing food, though of smaller amplitude.^183^ Interestingly, ablating these neurons caused death in ABA mice due to less mobilization of fat stores, leading to less circulating free fatty acids for energy, but it remains unknown how this relates to the altered activity shown by fiber photometry. In a subsequent study, AgRP neuronal activity was tracked across days using fiber photometry to reveal that ABA mice have an abnormally blunted inhibitory response to food compared with other food-restricted mice, only during the first three days of the ABA protocol.^184^ Interestingly, chemogenetic activation of AgRP neurons, which is aversive in the absence of food, when done in the presence of food is capable of reversing deficits in ABA mice such that they eat more and are less active. Taken together, the results strongly suggest that the hypoactivity of AgRP hypothalamic neurons may underlie some of the symptoms of anorexia. Interestingly, females with anorexia have been found to have elevated AgRP levels in their plasma,^185^ perhaps consistent with a role for AgRP in regulating fat mobilization peripherally in the setting of caloric restriction. The central mechanisms involving AgRP for anorexia in humans remain to be clarified.

### 4.4 Other Psychiatric Disorders

Please see the recent review by Gergues et al.^186^ for a review of findings from calcium imaging-based techniques relevant to mood disorders, anxiety disorders, schizophrenia, obsessive-compulsive disorder, and autism spectrum disorder.

## 5 Future Directions for Research and the Treatment of Psychiatric Disease

Though these studies have been greatly influential in our understanding of neural activity during appetite and aversive states, studies relying on behaviors as readouts for understanding aversive state encoding are somewhat limited by their reliance on behaviors for inferring underlying states. Population imaging studies have demonstrated that motor signals are a dominant pattern throughout the brain,^57^ and interestingly in the frontal cortex, during a task in which mice licked for rewards, licking-related activity dominated neural responses, obscuring underlying encoding of reward-predicting cues.^58^ By comparing licking during different reward conditions, the authors were able to disentangle reward encoding from motor output, highlighting the importance of disentangling behavior from the encoding of appetitive and aversive information. A similar approach was taken by Biane et al.,^154^ in which neural encoding was compared as mice either escaped from a threat or ran toward a reward, thus creating equivalent motor states aligned with distinct internal states. Comparing the encoding of different behaviors that reflect similar internal states has also been a powerful approach.^114^ Likewise, using aversive and appetitive stimuli to induce internal states raises the challenge of disambiguating sensory processing from internal state regulation, which has been addressed both with statistical modeling^127^ and using sensory stimuli of different sensory modalities that induce the same internal states.^130^ Finally, the simultaneous tracking of physiological metrics of internal states has illustrated internal state fluctuations that occur within the same behavioral states,^187^ highlighting the importance of multidimensional data acquisition to fully understand internal state representations.

To continue to translate discoveries on the circuit regulation of internal states for the understanding and treatment of human psychiatric disease, further studies determining how circuit hubs for internal state regulation are altered in human psychiatric disease will be necessary.^188^^–^^190^ In particular, understanding patterns of altered functional connectivity associated with dysfunctional internal state regulation may provide insights into targets for non-invasive interventional approaches^9^ such as transcranial magnetic stimulation (TMS)^191^ or transcranial direct current stimulation (tDCS).^189^^,^^192^ It seems likely that the internal state of patients will influence the plastic changes induced by TMS, and thus, further studies manipulating internal states or leveraging naturally occurring internal states would be of great benefit.^190^ A reasonable hypothesis is that brain areas and small or extended brain networks engaged during different internal states will exhibit preferential changes in brain plasticity if repetitive brain stimulation occurs during these states.^189^ Internal states—such as attention, arousal, and mood—modulate the brain’s response to repetitive brain stimulation.^189^^,^^193^ These internal states may enhance or suppress plasticity in neural circuits depending on the underlying brain activity during stimulation. For example, sensory areas may be more responsive during focused attention, whereas emotional centers such as the amygdala may be active in stress or anxiety.^194^ Repetitive brain stimulation may be more likely to promote plasticity when the brain is in high arousal states but has diminished effects during restful states.^195^ In this way, aligning brain stimulation with an individual’s internal state could optimize the effectiveness of treatments such as TMS or tDCS.^193^^,^^195^^,^^196^

An approach for hypothesis generation about this relationship could involve evaluating the activity of brain regions during different internal states to identify states that evoke activity in brain regions found to have diminished function activity in individuals with a specific psychiatric disease. Alternatively, pretreatment functional imaging correlates could be identified for responders versus non-responders to TMS. For instance, Downar et al.^197^ found that individuals less likely to respond to TMS targeted to the dmPFC exhibited diminished functional connectivity in reward circuits, and thus, attempts could be made to activate these circuits using external stimuli to induce a hedonic internal state that activates those regions, which may be beneficial for potentiating connections between these brain structures.^189^^,^^190^ Preclinical studies utilizing brain stimulation in rodents during different internal states may be informative for testing these ideas in advance of human trials.

## 6 Conclusion

Population imaging approaches have greatly expanded our capacity for understanding both microcircuits and interregional communication within the brain. As it has become increasingly clear that neural ensembles are a functional unit of computation within the brain, the capacity to record from many neurons simultaneously and to compare the activity of genetically defined cell types is particularly important for understanding neural representations. Understanding the neural representation and regulation of internal states is particularly relevant for understanding psychiatric disease, which almost universally involves dysfunctions in emotional regulation. Internal states represent a primitive basis for emotions in humans, and by understanding circuit motifs underlying the regulation of these states, we can greatly expand our understanding of emotional regulation circuits in humans. We anticipate that new insights about the neural regulation of internal states will be able to translate into improved treatment of human psychiatric disease, particularly using non-invasive brain stimulation.

## Data Availability

This paper does not present any original data. Any data cited would need to be provided by the original sources.
